# Celiac Male’s Gluten-Free Diet Profile: Comparison to that of the Control Population and Celiac Women

**DOI:** 10.3390/nu10111713

**Published:** 2018-11-08

**Authors:** Teba González, Idoia Larretxi, Juan Carlos Vitoria, Luis Castaño, Edurne Simón, Itziar Churruca, Virginia Navarro, Arrate Lasa

**Affiliations:** 1 Instituto de Investigación Sanitaria Biocruces Bizkaia, Hospital Universitario Cruces, UPV/EHU, CIBERDEM, CIBERER, 48903 Barakaldo, Spain; teba.gonzalez@gmail.com (T.G.); jcvitoria45@gmail.com (J.C.V.); lcastano@osakidetza.eus (L.C.); 2 Gluten Analysis Laboratory of the University of the Basque Country, Department of Nutrition and Food Science, Faculty of Pharmacy, University of the Basque Country (UPV/EHU), Paseo de la Universidad, 7, 01006 Vitoria-Gasteiz, Spain; ilarretxi@hotmail.com (I.L.); edurne.simon@ehu.eus (E.S.); arrate.lasa@ehu.eus (A.L.); 3 Servicio de Gastroenterología Pediátrica, Hospital Universitario Cruces, UPV/EHU, 48903 Barakaldo, Spain

**Keywords:** celiac disease, gluten-free diet, diary recommended intake, food habit, body composition

## Abstract

The aim of the present work was to analyze the body composition and dietary profile of Spanish celiac men and to compare them to control men and celiac women from our previous studies. Forty-two celiac men (31.5 ± 11.9 years) were recruited and anthropometric measurements were taken. Analysis of energy consumption, macro- and micronutrient intake and food frequency consumption was carried out. Celiac men were more overweight and obese than celiac women, but less than the control population, reporting the same energy intake and macronutrient distribution. Most micronutrient deficiencies in celiac men were not directly related to a gluten free diet; these were also observed for the entire population. The least adherence to Dietary Reference Intakes in women was reported for iron, iodine, potassium and selenium, whereas magnesium intake was higher than in men. Among celiac participants (both genders), cereal, vegetable and legume consumption was poor and meat intake was contrastingly excessive. In conclusion, the dietary profile of celiac men is as unbalanced as that of control men but slightly more than that of celiac women. General nutritional education should be given to both general and celiac populations, and specific advices to celiac men, in order to decrease the risk of celiac disease-related pathologies.

## 1. Introduction

Celiac disease (CD) is described as a permanent intolerance to gluten and is the most common chronic intestinal disease in Europe. Its estimated prevalence in Europe is around 1%, and this only refers to those that are diagnosed, since a significant number of patients have not been diagnosed yet [1,2].

This intestinal disease is more frequent in women than in men in a 2:1 ratio [3] and thus, in the vast majority of studies carried out in celiac people, it is common to find a larger number of female participants than males. Moreover, it must be pointed out that, in general, the male population is less likely to take part in health promotion programs than women are. Different reasons for this have been put forward, such as their low recruitment, delayed help-seeking behavior, and less interest and knowledge about health-related topics and habits [4,5]. As a result, this population cohort is often undervalued and does not always receive gender-specific healthcare.

The only effective treatment for celiac disease is a strict lifelong gluten-free diet (GFD). In fact, small amounts of gluten ingestion can cause important damage-causing disorders in the intestinal mucosa. Apart from gluten absence, a GFD must guarantee nutritional balance and so prevent deficiencies. However, when the nutritional composition of GFD of celiac people has been assessed, imbalanced proportions of macronutrients and several deficiencies in vitamin and mineral content have been observed [6,7,8,9,10,11]. Specifically, a GFD is usually accompanied by a low intake of carbohydrates, iron, calcium, folate, niacin, zinc and fiber and excess of saturated fats.

Some aspects of the GFD profile could be linked to a higher risk of several diseases. For instance, the relationship between low fiber, high saturated fat intake and cardiovascular diseases and obesity has been widely described [12,13]. Micronutrient deficiencies are related to comorbidities such as ferropenic anemia and osteopenia. This fact enhances the importance of complying with not only the intake recommendations of some key nutrients such as iron, calcium, vitamin D, but also those of other important molecules that regulate the immune system and help metabolic status to be balanced (zinc, magnesium, selenium, folate and so on), either in women or in men. In fact, it has been described that these deficiencies persist in some of these patients, even if they follow a strict GFD [8,10,14,15].

Bearing in mind all the above mentioned, the aim of the present work was to evaluate the body composition of adult celiac men from a region in Spain and the nutritional composition of the GFD they followed, as well as compare it with international recommendations, and with dietary habits of the general population (Spanish adult men). As a second objective, energy and nutrient intake and dietary habits of celiac men were compared to those reported in our previous studies for celiac women from the same region.

## 2. Materials and Methods

### 2.1. Participants and Procedure

The present study used data from a celiac men cohort recruited between 2007 and 2013 from three regions of the Basque Country (Araba, Gipuzkoa, and Bizkaia), in the north of Spain, as part of a prospective SUSFOOD study conducted in collaboration with the Gastroenterology and Endocrinology Units of Cruces University Hospital and Coeliac Association from the Basque Country. Forty-two celiac men took part in the study (mean age ± SD: 31.5 ± 11.9); all participants were diagnosed with celiac disease according to ESPGHAN guidelines and followed a GFD for at least one year. Exclusion criteria included a history of chronic diseases such as cardiovascular disease, diabetes, hyperthyroidism/hypothyroidism, hypercholesterolemia, hypertriglyceridemia or high blood pressure levels, other digestive pathologies that need specific dietary advice, and lack of motivation to participate in the study. Written informed consent was obtained from all participants, after receiving information about the survey. This study was approved by the Ethical Committee of Cruces Hospital (CEIC E-08/66) and the Ethical Committee of the University of The Basque Country (CEISH/76/2011).

Celiac women were recruited simultaneously and their data were collected in the same way as results from men; in fact, the woman cohort is part of the SUSFOOD study mentioned, which has already been published [6]. Control men data were obtained from the ENIDE nutritional survey carried out in Spain, based on 1589 adult men and conducted over the same period of time as the present work (ENIDE).

### 2.2. Anthropometric Measurements

Anthropometric measurements were collected by trained personnel. Body weight (±10 g) was measured after voiding using a digital integrating scale (SECA 760). Height was determined to the nearest 5 mm using a stadiometer (SECA 220). Body Mass Index (BMI) was calculated from weight and height (kg/m^2^). The BMI values were categorized according to the World Health Organization (WHO) criteria as follows: Below 18.5 kg/m^2^ considered as underweight, 18.5–24.9 kg/m^2^ as normal weight, 25–29.9 kg/m^2^ as overweight and >30 kg/m^2^ as obese (WHO).

### 2.3. Body Composition and Energy Expenditure

Fat mass was estimated by a direct segmental multiple-frequency bioelectrical impedance analysis method (Inbody 230; Biospace, Seoul, Korea). Two skin electrodes were placed on the feet and two on the hands. Following the standard procedure, whole-body resistance and reactance were measured. Fat mass was evaluated from total-body impedance (Z). The guidelines of Gallagher et al. were used as reference for body fat mass [16].

Weight, height and age were used to calculate individual energy expenditure according to the Harris-Benedict formula. Standard activity level value was applied.

### 2.4. Dietary Assessment

Dietary intake was assessed using 3-day 24-h food recalls (24 HR), two on weekdays and one at the weekend. Sixteen participants filled out a food frequency questionnaire (FFQ). Trained nutritionist-dieticians recorded the answers of participants. Food portions and amounts were determined by using photographs of rations and sizes described in the Photo Album, as per the author of [17]. Energy and nutrient intakes were calculated by the nutritional software program “Alimentación y Salud” (AyS, Software, Tandem Innova, Inc., Huesca, Spain). The composition of specific gluten-free products for celiac people consumed by the participants was collected from the manufacturers and added into the food composition database of the program before calculations. As gluten-free product labels did not indicate micronutrient content (vitamins and minerals), an estimation with homologous gluten-containing products was carried out.

Dietary reference intakes (DRI) for Spanish population issued by the Spanish Societies of Nutrition, Feeding and Dietetics (FESNAD) in 2010 were taken as references for the interpretation of the 24 HR [18]. In the case of FFQ, Spanish Society of Community Nutrition (SENC) recommendations were used for the correct interpretation of the results [19].

Moreover, the results were compared to energy, nutrient and food intake of celiac women [6] and those of the mentioned Spanish reference population (ENIDE) [20].

### 2.5. Statistical Analysis

Statistical analyses of results were performed by using the IBM SPSS statistical program, version 23 (IBM Inc., Armonk, NY, USA). Normality in the distribution was assessed by the Kolmogorov-Smirnov test, and homogeneity by Levene’s test. Statistical analyses were performed in order to calculate differences between celiac men and control population were performed with summary *t* Student’s test, and those between celiac men and celiac women with Chi-square test. *p* values < 0.05 were accepted as significant.

## 3. Results

### 3.1. Anthropometric Measurements

Anthropometric data of celiac men from the present study are shown in Table 1.

57.1% of the participants showed normal BMI values and only 4.8% were underweight. In contrast, 38.1% of them were above normal BMI values, 26.2% were overweight, and 11.9% obese. Fat mass measurements indicated that 52% of the participants had excessive adiposity and 41% were between normal values. Only three participants (7%) had very low fat mass values.

### 3.2. Dietary Intakes

#### 3.2.1. Energy, Macronutrients, Fiber and Cholesterol Intake in Celiac Men

**Figure 1:**
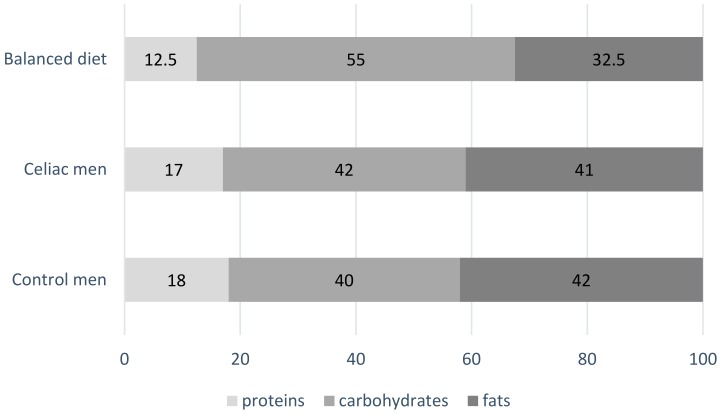
Mean contribution of macronutrients to energy in Spanish celiac (*n* = 42) and control men (*n* = 1589) (ENIDE study, representative at national level of the adult population) compared to the recommended contribution in a balanced diet proposed by the Federation of Spanish Societies of Nutrition and Dietetics (FESNAD).

Daily energy intake was comparable to that observed in control men in ENIDE study. Energy distribution among macronutrients was not balanced in celiac men. To be specific, proteins and fats were consumed in excess (17% and 41% respectively) accompanied by a small amount of carbohydrates (42%) (Figure 1). When these data were compared to those of the ENIDE study, no significant differences were observed in energy and macronutrient consumption.

In order to evaluate fat sources, dietary lipid profile was calculated. While saturated fatty acid (SFA) and monounsaturated fatty acid (MUFA) consumption was similar in celiac men and control men, polyunsaturated fatty acid (PUFA) consumption of celiac men was lower. In general terms, saturated and unsaturated fatty acids ratios were reached, as in the ENIDE survey (Table 2) [21]. Cholesterol ingestion was also similar in both groups, higher than that recommended [18].

Regarding dietary fiber consumption, celiac men were below recommendations (25–35 g/day). In fact, fiber consumption of 28% of participants was below 15 g per day and that of 43% between 15.1 and 25 g per day. A similar intake of fiber was also reported in control men, taken from the ENIDE survey (Table 2).

A comparison of energy intake between celiac men from the present study and celiac women from our previous studies [6] revealed that while the majority of women (65%) consumed the correct amounts of calories in their diet, less than half the men (46%) did so (Table 3). Moreover, only 6% of women consumed calories in excess, which, by contrast, 14% of men did. However, macronutrient distribution, similar in both genders, was higher than recommendations for proteins and fat intakes, and lower in the case of carbohydrates.

Dietary lipid profiles were similar between celiac men and women. In both groups, the highest percentage of participants consumed excessive SFA and cholesterol, though even more so in men.

Although both groups contained a high proportion of subjects with low fiber intakes (71% in men vs. 96% in women), there were more men that achieved adequate fiber consumption (26% in men vs. 4% in women).

#### 3.2.2. Micronutrients Intake in Celiac Men

When vitamin and mineral mean intake of celiac participants was compared to that of control men, differences in eight micronutrient mean consumption were observed (Table 4). Celiac men consumed lower amounts of vitamin E, niacin and magnesium than the control group. By contrast, their riboflavin, B6, zinc, potassium and selenium mean intake was higher.

A 67% (2/3) DRI cutoff value for deficient micronutrient intake was set as reported by the literature [22,23]. According to this cutoff, vitamin A, D and E, iodine, folate and magnesium were deficient, as a small amount (41–81%) of participants accomplished it (Table 5). In the case of calcium, zinc, selenium and vitamin C, most participants (around 90% of them) fulfilled 2/3 of the DRIs. None of the celiac men showed low intakes of vitamin B6, B12, niacin, riboflavin, thiamine, phosphorus and iron.

Several differences were found in mineral intake accomplishment, but not in vitamins, when comparing celiac men and women habits (Table 5). Celiac women fulfilled magnesium requirements better than celiac men and, by contrast, iron, iodine, potassium, and selenium DRIs were better complied by celiac men.

### 3.3. Food Consumption Frequency of Celiac Men

**Figure 2:**
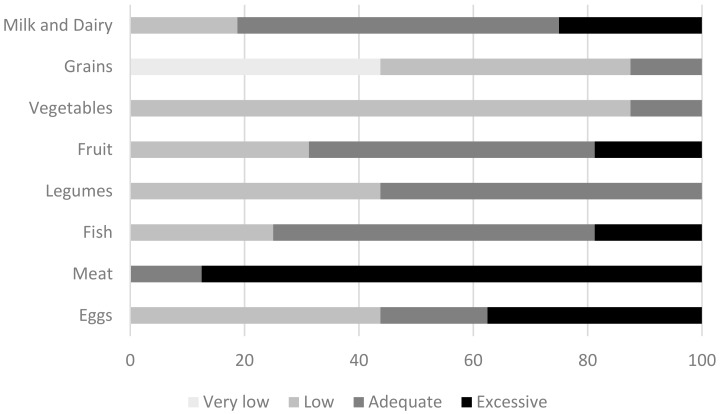
Compliance of food frequency consumption in celiac men by servings per day or week, according to the Spanish Society of Community Nutrition (SENC).

Figure 2 summarizes main food group consumption frequency. Cereal consumption data indicated that only 13% of celiac men fulfilled these food group recommendations, which means at least four servings per day (Figure 2). Moreover, almost half of them (44%) consumed a small or a very small amount of cereals (fewer than two servings) per day.

The vast majority of participants (84%) did not reach vegetable consumption recommendations (10 portions/week) [19]. One-third of celiac men did not consume the minimum recommended two servings of fruit daily. Furthermore, legume consumption was also low in 42% of participants, which means that they consumed less than two portions of pulses per week.

With regard to animal origin food consumption, almost half of the participants fulfilled the recommendations of dairy products, 2–3 servings per day, whereas 25% reported an excessive consumption, which means more than 4 dairy servings per day. Only 19% of celiac men achieved egg consumption recommended by the SENC, while 38% had an excessive consumption. The ingestion of fish and derivatives was sufficient in nearly half of celiac men and almost 20% of participants consumed it in excess. By contrast, meat consumption was excessive in the vast majority of the subjects (88%), which means that they ate more than 4–5 servings of meat per week.

In relation to the foods considered for occasional consumption, it must be pointed out that celiac men followed the recommendations (data not shown). Participants used olive oil, which is rich in monounsaturated fatty acids, as their fat source, and discarded other lipid sources such as margarine or butter as their main lipid source for cooking.

Finally, the analysis of food frequency questionnaires in celiac men and celiac women revealed similar results. No differences were found in dairy products, grains, vegetables, fruits and meat consumption. However, when fish and egg consumption were evaluated, a tendency toward higher percentage of celiac men with excessive consumption of these foods was observed, compared to celiac women. Concretely, 19% of men consumed fish in excess vs. 6% of women (*p* = 0.09). Moreover, 37% of men consumed eggs in excess, whereas 17% of women did so (*p* = 0.08).

## 4. Discussion

Male participation in health promotion programs has usually been lower than that of women, probably due to their lower interest in health-related topics, among other reasons [4,5]. The studies in the literature comparing men and women’s dietary habits provide different outcomes, attributable to differences in methodologies, population sizes, regional habits, and recommendations. Some studies described different patterns of deficiencies either in macro- or micronutrients between genders [24,25]. In the case of celiac disease, even though clinical trials have been published with both female and male participants, the recruited number of men is usually small. Thus, evidence about celiac men’s body composition, diet quality, and eating pattern is rather scarce. The present study presents detailed energy and nutrient intake and dietary habits of a meaningful celiac male group, comparing them to a male control population and to celiac women from the same geographical region.

Obesity and overweight are rising among celiac patients, which is increasingly of concern to clinicians [11,26]. In this study, anthropometric data revealed that celiac men showed an alarming 38.1% of overweight and obesity, while 52% of participants showed an excessive fat mass. Nevertheless, this prevalence was lower than that observed in the control population [27,28]. These results were consistent with other studies, where celiac men registered similar BMI index values [8,29,30] which were also lower than those of the control populations [6,7,31,32].

Comparing these results with data from women (only 7.4% of overweight in celiac women), major gender differences were found. Accordingly, the prevalence of obesity and overweight in general population is greater for men [33,34,35]. These differences could, as stated before, be due to the lesser interest that men pay to health status and its care [4,5]. Nevertheless, it must be pointed out that in the case of celiac patients, most studies do not find gender differences in this parameter [8,29]. However, Tucker et al. (2012) [36] found that females were more likely to be obese, when BMI reaches values of 30 or above.

Even though energy intake of celiac men was similar to that of the control and to celiac men from other studies [8,20,29,30], our data revealed that 14% of celiac men consumed calories in excess, more than 120% of their energy expenditure, and 24% of them exceeded 110% of requirements. These results were not found in women, whose energy intake was suitable in 65% of the participants. All of the above explains the high percentage of obesity found among male participants from the present study. By contrast, 40% of the participants were under 80% of their energy expenditure, reflecting a possible underreporting, as found by others [29,37].

Celiac men showed imbalanced energy distribution, similarly to previous studies, but it must be pointed out that participants in this study consumed even fewer carbohydrates and more fat than those of earlier studies [29,30]. However, the macronutrient consumption of Spanish celiac women and the control population was similar to that observed in the male celiac group studied. Thus, it seems that imbalanced energy distribution is not GFD-related but could be associated to geographical dietary habits. Of these, low cereal and vegetable consumption and excessive meat intake, observed in both men and women [6], as well as in control men [38], could be the main contributors to the macronutrient imbalance.

With regard to dietary fat sources, SFA, MUFA and cholesterol were consumed in excess, although adequate fatty acid ratios were reached. These results were similar to those observed in control population, reinforcing the idea of this not being a GFD-related imbalance. The excessive consumption of meat (88% participants) and eggs (37%) could justify the results observed. However, the proportion of men eating excessive SFA and cholesterol was significantly greater than that of women. Although meat consumption did not differ between men and women, the excessive consumption of eggs among celiac men could impair these parameters. By contrast, intake of PUFAs was slightly lower in the male celiac population than in control men. However, 90% of celiac men participating showed adequate PUFA intake, more than women did. The higher fish consumption observed in men could be on the basis of this difference.

As far as fiber is concerned, celiac male consumption was low, similar to other studies conducted with celiac men and control population [8,20,29,30]. The low consumption of vegetables, legumes and grains could explain, at least in part, this outcome. However, fiber recommendations were more obeyed by celiac men than by celiac women, due to higher general food intake and thus greater amounts of plant origin foods.

The celiac population has been associated with an increased cardiovascular risk [39,40]. Although factors influencing the association between CD and cardiovascular disease could be related to the pathophysiology of CD, there is no doubt that the body composition and dietary pattern of celiac men described, with high intake of deleterious components, such as SFA, and low intake of protective ones, such as fiber, play an essential role.

As stated previously, micronutrients have also been a matter of concern for the celiac population. In our study, some differences in micronutrient mean intake arose between celiac men and their control. Vitamin E, niacin and magnesium was lower in celiac men than in control men and, by contrast, that of riboflavin, B6, zinc, potassium and selenium was higher, pointing to a different dietary pattern of this collective. While mean intakes provided some important information, it was necessary to analyze to what extent this population was below micronutrient recommendations, in order to identify possible deficiencies and health-risks.

The percentage of celiac men who complied with micronutrient DRI varied among nutrients, and also between genders. The most alarming deficiencies reported in this work for celiac men were those of vitamin D, vitamin E, and iodine, all of them with less than 50% of individuals fulfilling two thirds of DRI.

Wild et al. (2010) also reported vitamin D deficient intake in celiac men (no more than 20% of patients fulfilled the DRI) [8,29,30]. These data were also in accordance with the general population [8,20,29,30]. Vitamin E and iodine mean intakes were also low for both celiac and control men. In particular, vitamin E was even lower in celiacs, where only 48% reached the 67% of DRI. Iodine and vitamin E are not usually analyzed in GFD assessments, but considering their extremely low intake, specific nutritional advice, and probably supplementation, should be considered for the celiac male population.

In addition to the aforementioned important deficiencies, other micronutrients, such as folate and magnesium, were also below recommendations in celiac men (less than 80% achieved two-thirds of DRI). These data were in agreement with previous studies from the literature [8,30,37].

The comparison between celiac men and women in their adherence to vitamin and mineral recommendations revealed differences only in some mineral intakes. Less fulfilment of DRIs in women was reported for iron, iodine, potassium, and selenium, whereas magnesium intake was higher than that of men. This could be due to the lower energy intake of women [6], which could hinder the achievement of some mineral intakes. Iron was a special case, because men fulfilled totally the DRIs, but only 69% of women achieved 2/3, due to more demanding requirements of this mineral at some life stages for them. Considering the high prevalence of anemia in patients with CD, this is an important issue to be addressed in women.

In general, it can be stated that micronutrient deficiencies in celiac men were not directly related to a GFD, and were concerning for the entire population. Only magnesium and vitamin E intake appeared to be lower than the control, and below recommendations for both groups in the case of vitamin E. Folate, iodine and vitamin D intakes were the most worrying, giving the anemia, thyroid disease and osteopenia prevalence of this collective [41,42].

One of the most cited reasons for micronutrient deficiency in a GFD is gluten-free products (GFP) composition. In fact, gluten-containing grains and foods are a major source of micronutrients [43,44], and often GFP do not contain the same levels of micronutrients as their counterparts—e.g., thiamine, riboflavin, niacin, folate, vitamin D, calcium, or iron [10,45,46,47,48]. Instead of those GFP, higher gluten-free whole cereals (amaranth, sorghum, millet, etc.) and pseudocereals (quinoa, buckwheat, etc.) are more interesting, as they contain the fiber and micronutrients necessary to balance [11,49]. Nevertheless, fortification of gluten-free flours and GFP could be also of interest, but more research is necessary in order to correctly identify which nutrients are suitable for general fortification and which for personal supplementation.

For this purpose, all micronutrients should be addressed in the studies, and the micronutrient composition of GFP, which needs to be studied in depth, should be incorporated into the databases. Selenium, iodine and vitamin E intakes are scarcely analyzed in the literature, and they are still important to ensure the health status of celiac people.

The main limitation of the present study was that micronutrient intake coming from GFP was calculated according to the micronutrient content of their gluten-containing counterparts. Thus, data from the present work could represent an overestimation of the real consumption of vitamins and minerals. Moreover, people participating in this kind of studies are more concerned about health issues and self-caring, which could also have overestimated the results. Furthermore, the experimental design did not include a control group of men, and people with comorbidities were excluded from the study. Nevertheless, this is the first study where dietary habits of a cohort of Spanish celiac men were compared to those of the control population and to those of Spanish celiac women. It is worth noting that the sample size was higher than those used in other studies with celiac men.

In summary, inadequacies in terms of both, macro- and micronutrients, in celiac male diets are found. Some of them also appear in the diet of the control population, pointing to unsuitable habits of the entire population, but other changes are gender-specific and fewer GFD-dependent. Effective general recommendations for the global male population should be given, as well as specific advice for celiac people, principally regarding micronutrient intake. Greater consumption of gluten-free cereals or pseudocereals, vegetables, and legumes and less of that of meats should be recommended. Personalized dietary advice and long-term follow-up for celiac people, in particular for celiac men, could contribute towards preventing nutrient deficiencies related to dietary imbalance and to obtaining a better health status, lowering the risk of CD-related pathologies.

## Figures and Tables

**Table 1:** Characteristic of celiac participants included in the survey.

Characteristic	Men
*N*	42
Age (year)	31.5 ± 11.9
Mean duration of GFD (year)	20.9 ± 11.9
Height (cm)	176.2 ± 6.2
Weight (kg)	75.8 ± 13.9
Fat mass (%)	24.1 ± 8.1
**Body-Mass Index**	
Mean (kg/m^2^)	24.7 ± 4.1
Underweight < 18.5—no. (%)	4.8
Normal 18.5–24.9—no. (%)	57.1
Overweight 25–29.9—no. (%)	26.2
Obese > 30—no. (%)	11.9

Notes: Values are mean ± SD or percentages; SD, standard deviation; *N*: sample size; no, number of subjects; GFD, gluten-free diet.

**Table 2:** Energy and nutrient distribution in celiac and Spanish control men and results from recent studies.

Characteristic	Celiac Men (*n* = 42)	Spanish Men (ENIDE Study) (*n* = 1589)	*p* Value	Sheperd 2013 (11 Men)	Martin 2013 (18 Men)	Wild 2010 (31 Men)
Energy (kcal)	2355.4 ± 659.0	2546.8 ± 860.9	NS	2697 ± 445	2401 ± 592	2500 ± 717
Protein (g)	100.2 ± 30.1	109.4 ± 47.7	NS	98.8 ± 22.83	92.4 ± 17.5	92.7 ± 24.9
Carbohydrate (g)	220.7 ± 84.7	242.7 ± 101.8	NS	294 ± 72.3	267 ± 95	315.6 ± 98.5
Fat (g)	114.0 ± 32.2	114.9 ± 46.0	NS	67.9 ± 50.58	97.4 ± 29.7	93.2 ± 36.6
MUFA (g)	52.4 ± 16.1	48.29 ± 22.2	NS	-	-	-
PUFA (g)	13.2 ± 5.8	15.5 ± 8.5	<0.05	-	-	-
SFA (g)	34.5 ± 11.2	32.71 ± 15.55	NS	34.7 ± 7.7	-	-
(PUFA + MUFA)/SFA	1.93	1.95	-	-	-	-
PUFA/SFA	0.38	0.47	-	-	-	-
Cholesterol (mg)	421.2 ± 132.8	423.82 ± 181.25	NS	-	-	-
Fiber (g)	20.3 ± 7.1	20.94 ± 11.38	NS	30.2 ± 7.7	22.3 ± 6.0	13.7 ± 5.3

Notes: Values are means ± SD; Spanish adult men data were taken from the Spanish dietary nutritional assessment (ENIDE study, representative of the adult population at national level); SD, standard deviation; NS: non-significant; PUFA, polyunsaturated fatty acids; MUFA, monounsaturated fatty acids; SFA, saturated fatty acids.

**Table 3:** Percentage of celiac men (*n* = 42) and women (*n* = 54) that fulfilled energy and macronutrient recommendation and their comparison.

	Recommended Intake *	Celiac Men			Celiac Women			*p* Value
					[6]			
		Low	Adequate	Excessive	Low	Adequate	Excessive	
Energy intake	±20% of EE	40	46	14	30	65	6	<0.001
Protein	10–15%	0	19	81	0	20	81	NS
Carbohydrate	50–60%	98	2	0	91	9	0	NS
Total Fat	30–35%	0	5	95	4	11	85	NS
SFA	<10%	0	17	83	0	33	67	<0.05
MUFA	15–20%	14	31	55	31	37	28	NS

Notes: * Recommended energy and nutrient intake in a balanced diet proposed by the Federation of Spanish Societies (FESNAD). EE: energy expenditure; SFA, saturated fatty acids; MUFA, monounsaturated fatty acids; NS: non-significant. *p* value corresponds to differences between celiac males’ and females’ suitable intakes.

**Table 4:** Micronutrients mean intake in celiac and Spanish men.

	Celiac Men (*n* = 42)	Control Men (ENIDE Study) (*n* = 1589)	DRI: FESNAD (2010)	*p*
				Celiac Men vs. Spanish Men (ENIDE Study)
Vitamin A (ug)	802 ± 340	748 ± 338	700 ^a^	NS
Thiamin (mg)	2.0 ± 1.4	2.1 ± 6.7	1.2 ^b^	NS
Riboflavin (mg)	2.2 ± 1.1	1.5 ± 0.8	1.6	<0.001
Vitamin B6 (mg)	2.7 ± 0.9	2.0 ± 0.9	1.5 ^c^	<0.001
Vitamin B12 (ug)	8.1 ± 5.6	7.9 ± 6.1	2	NS
Vitamin C (mg)	143 ± 82	131 ± 81	60 ^d^	NS
Vitamin D (ug)	4.4 ± 4.5	4.3 ± 4.5	5 ^e^	NS
Vitamin E (mg)	10.8 ± 5.1	14.9 ± 8.4	15	<0.001
Niacin (mg)	38.5 ± 13.6	45.7 ± 39.5	18 ^f^	<0.01
Folate (ug)	302 ± 115	296 ± 121	300	NS
Calcium (mg)	939 ± 295	886 ± 345	900 ^g^	NS
Iron (mg)	16.5 ± 5.1	16.1 ± 6.5	9 ^h^	NS
Magnesium (mg)	323 ± 107	396 ± 139	350	<0.001
Iodine (ug)	117 ± 88	100 ± 50.5	150	NS
Phosphorus (mg)	1580 ± 442	1535 ± 471	700 ^i^	NS
Zinc (mg)	12 ± 4.0	10.5 ± 3.7	9.5 ^j^	<0.01
Potassium (mg)	3481 ± 980	3045 ± 917	3100	<0.01
Selenium (ug)	93.8 ± 53.5	63.5 ± 35.1	55	<0.001

Notes: Values are means ± SD. Spanish adult men data were taken from the Spanish dietary nutritional assessment (ENIDE study, representative at national level of the adult population). SD, standard deviation; DRI, dietary reference intake; FESNAD, Federation of Spanish Societies of Nutrition and Dietetics; NS: non-significant. ^a^ Vitamin A, 800 mg for 16–19 year range men; ^b^ Thiamin, 1.1 mg for >60 years old men; ^c^ Vitamin B6, 1.4 mg for 16–18 years old and 1.6 mg for >60 years old men; ^d^ Vitamin C, 70 mg for >60 years old men; ^e^ Vitamin D, 7.5 mg for >60 years old men; ^f^ Niacin, 17 mg for 50–69 years old men; ^g^ Calcium, 1000 mg 16–19 years old and for >60 years old men; ^h^ Iron, 10 mg for >60 years old men; ^i^ Phosphorus, 800 mg for 16–19 years old men; ^j^ Zinc: 10 mg for >60 years old men.

**Table 5:** Percentage of celiac men (*n* = 42) and women (*n* = 54) that fulfilled at least 2/3 of DRI (FESNAD, 2010) and their comparison.

	Celiac Men	Celiac Women	*p* Value
		[6]	
	% of Participants that Fulfilled at Least 67% of Recommendations	% of Participants that Fulfilled at Least 67% of Recommendations	
Vitamin A (ug)	81	89	NS
Thiamin (mg)	98	100	NS
Riboflavin (mg)	98	98	NS
Vitamin B6 (mg)	100	100	NS
Vitamin B12 (ug)	100	100	NS
Vitamin C (mg)	93	96	NS
Vitamin D (ug)	45	52	NS
Vitamin E (mg)	48	61	NS
Niacin (mg)	100	100	NS
Folate (ug)	76	82	NS
Calcium (mg)	86	87	NS
Iron (mg)	100	69	<0.001
Magnesium (mg)	71	91	<0.05
Iodine (ug)	50	20	<0.01
Phosphorus (mg)	100	100	NS
Zinc (mg)	91	98	0.093
Potassium (mg)	98	85	<0.05
Selenium (ug)	93	69	<0.01

Notes: Recommended intake of vitamins and minerals proposed by the Federation of Spanish Societies (FESNAD). NS: non-significant. *p* value correspond to differences between celiac men and celiac women’s appropriate intakes.
